# Efficacy of the adapted Cycles approach in telepractice speech-language pathology with a parental focus for children with speech sound disorders

**DOI:** 10.1590/2317-1782/e20240216en

**Published:** 2025-08-11

**Authors:** Daniela Aparecida Barbosa, Haydée Fiszbein Wertzner

**Affiliations:** 1 Departamento de Fisioterapia, Fonoaudiologia e Terapia Ocupacional, Faculdade de Medicina, Universidade de São Paulo – USP - São Paulo (SP), Brasil.

**Keywords:** Speech Sound Disorder, Teleconsultation, Telemonitoring, Child Language, Speech Therapy, Gamification, Speech Perception, Parents

## Abstract

**Purpose:**

To evaluate the effectiveness of the adapted Cycles approach to telepractice and family engagement in the treatment of children with speech sound disorder (SSD).

**Methods:**

Ten children aged 5:0 to 7:8 years with a diagnosis of SSD participated. Two multiple baseline assessments and two post-treatment assessments were conducted. Effectiveness was measured by comparing pre- and post-intervention percentage of consonants correct (PCC), percentage of consonants correct revised (PCC-R), Process Density Index (PDI), and the number of phonological processes with occurrence >25% and the intervention effect size (ES). Family perceptions of daily training were obtained via digital interview and Likert scale. Subjects were randomly distributed to G1: two weekly online sessions with the speech therapist, and G2: half the sessions conducted by the caregiver trained by the speech therapist. Both groups received 12 sessions and performed daily speech training.

**Results:**

All participants showed increased PCC, PCC-R, and decreased PDI and number of phonological processes with occurrence >25%, with ES ranging from small to large. There was a trend towards statistical significance (ES G2 > G1). All caregivers reported high child interest in activities and ease of execution, with a positive family experience (Likert=4) training the child's speech using digital resources.

**Conclusion:**

The effectiveness of the proposed approach ranged from medium to high. The performance of the groups was similar, with a trend towards greater effectiveness for G2, which focused on family involvement. Parental training resulted in good engagement in sessions and daily training.

## INTRODUCTION

Speech sound disorders (SSD) are characterized by difficulties in motor production, auditory perception, and/or cognitive-linguistic skills, affecting the perception and production of speech sounds^(1)^. These disorders are among the most common in children, with an estimated prevalence between 2.3% and 24.6%^(1,2)^. In Brazil, the occurrence ranges from 8.26% to 20.63%^(3)^.

In typical language development, children are expected to gradually overcome phonological processes until approximately 7 years old, achieving speech without phonological simplifications^(4)^.

The phonological processes that characterize SSD are observed during typical phonological development and involve error patterns that simplify the phonological rules of the language^(4)^. Maintaining phonological processes beyond the expected age for their elimination is suggestive of SSD^(5)^.

The Cycles approach is based on the interaction between cognitive-linguistic, perceptive, and speech production processes^(6,7)^. The bases of the Cycles approach consider that phonological acquisition is a gradual process; speech sound acquisition occurs by listening to speakers; the association between kinesthetic and auditory sensations enables the automation of the acquired sound; the stimulation of new speech sounds facilitates their correct production; the child is involved in their phonological acquisition; phonological skills are generalized to other sounds learned spontaneously; phonological analysis is used to initiate treatment at a functional level for the child^(6)^.

In Brazil, the adapted Cycles approach is applied to children with incomplete phonetic inventory, phonological processes not expected for their age (with an occurrence greater than 25%), difficulties in auditory discrimination and production of some speech sounds, and difficulties in phonological awareness^(8)^.

Studies have addressed various forms of intervention more recently, including in-person and synchronous and asynchronous telepractice^(9,10)^.

### SSD-intervention via telepractice

Telepractice has allowed significant gains in the delivery of SSD-intervention to children and in the adherence of families to it, by overcoming the barriers related to distance and travel. Furthermore, serious digital games^(11-13)^, aimed at teaching certain concepts, keep the child engaged during the telepractice and interested in carrying out the daily training inherent to the treatment^(13)^. Thus, telepractice with serious digital games has the potential to provide intervention in a greater dose and intensity than traditional in-person interventions^(14)^.

Studies^(15,16)^ have already shown that both in-person and telepractice interventions for SSD are effective, with the additional advantage of online therapy facilitating intensive and daily practice. Despite the risk of problems with Internet connection and usability of technological resources, telepractice for phonological and articulatory interventions has validated efficacy for rural^(17)^ and school^(16)^ populations.

Besides technological resources, telepractice also depends on human resources. Therefore, before indicating this type of intervention, it is necessary to consider the speech-language pathologist’s (SLP’s) training to use digital resources and manage possible telepractice problems, as well as the family's digital literacy with a focus on communication and information^(18)^.

It is also very important to understand telepractice from the perspective of the children's parents and caregivers, since they play a crucial role in the treatment. The involvement of parents in in-person intervention of children with SSD is still poorly detailed in studies^(19)^. They are commonly included in daily training, to which they are previously prepared by observing the SLP’s procedures, practicing them under the professional’s supervision, and receiving written instructions. This movement has been expanding because of the clear need to decentralize the SSD-intervention from the professional and seek a more intense partnership with the families of children undergoing treatment^(20)^.

A recent study^(21)^ investigated the gains from increasing the frequency of speech-language intervention in 10 children with SSD and verified the percentage of consonants correct (PCC) and the parents’ perception of their children regarding the change in speech intelligibility and the degree of satisfaction with the treatment. The development of the implementation plan for increasing the frequency of the intervention had the collaboration of two parents of children with SSD, which may have contributed to the assertiveness of the new proposal for greater engagement of the other families participating in the research.

Given the need for greater knowledge regarding the use of telepractice in SSD interventions, the present study aimed to evaluate the effectiveness of the proposed intervention and the family's engagement in the application of the Cycles approach adapted to telepractice in the treatment of children with SSD.

## METHOD

This study was approved by the Research Ethics Committee of the School of Medicine at the University of São Paulo, under approval number 3,340,790 and CAAE no. 10019019.8.0000.0065. All participants’ parents signed an informed consent form. The subjects were previously instructed on the intervention process through an assent form, in which they indicated their assent by drawing or signing.

### Study population

The population consisted of 10 caregivers (father or mother) and their children, aged 5:0 to 7:11 years, residing in the cities of the Greater São Paulo, Brazil.

The inclusion criteria were being 5:0 to 7:11 years old; without previous treatment, or one completed/interrupted for at least 6 months; having Brazilian Portuguese (BP) as their native language; without complaints of cognitive, emotional, or neurodevelopmental alterations; having a PCC revised (PCC-R)^(22)^ lower than 93.4%^(23)^ in at least one of the phonology tests^(24)^ of the ABFW Child Language Test; being consistent in the Speech Inconsistency test^(25)^; having hearing thresholds within normal standards; belonging to a family nucleus with digital literacy focused on communication and learning.

Exclusion criteria were having any degree of hearing loss, diagnosed through audiological evaluation; other language, cognitive, or neurological impairments; not having a means of connecting to the Internet; or missing more than three alternate treatment sessions.

### Data collection

Participants initially underwent assessments to diagnose SSD (A1-A), multiple baseline assessments (A1-A and A1-B) to identify their natural development curve^(26)^, and reassessments (A2-A and A2-B) to verify the influence of SSD-intervention on this development. The child's speech samples obtained during the assessments were recorded on video with audio for later analysis.

The parents' perception of the speech training performed at home with the children was collected throughout the therapeutic intervention by completing an online interview with closed and open-ended questions.

### Multiple baselines (A1-A and A1-B)

A1-A was performed in two to three sessions to prevent the child's fatigue from interfering with their performance in the tests. The interview on the use of digital resources, audiological evaluation, and a battery of tests was applied as listed below:

- Phonological Sensitivity Test, auditory version (TSF-A, in Portuguese)^(27)^. It consists of four tests that verify performance in the skills of equal alliteration, different alliteration, equal rhyme, and different rhyme. Each test has 15 items, the first three for training and 12 for application and analysis.
- Speech Inconsistency Test (SI)^(25)^, to calculate the index of speech inconsistency. It has 25 pictures, named three times in different sequences, and interspersed with distracting activities. Each of the 25 words was analyzed, and if the three namings were the same, the production of the word was considered consistent.
- Phonology tests (word imitation and picture naming) from the ABFW Child Language Test^(24)^. The test was recorded on audio and video, and phonetic transcription was subsequently performed twice by the researcher to ensure reliability. The phonological processes were analyzed according to the guidelines of the ABFW Phonology test; those with more than 25% occurrence in an age range above that expected for overcoming were considered productive and targets for treatment. The number of different phonological processes with an occurrence greater than 25% was calculated.
- PCC^(28)^: refers to the percentage of consonants produced correctly in relation to the percentage of total consonants contained in the speech sample analyzed, with errors considered as phoneme substitution, omission, and distortion. For this index, the authors also classify the severity as mild (above 85%), mildly moderate (65% to 85%), moderately severe (50% to 65%), or severe (below 50%).
- PCC-R^(22)^: differs from the PCC in that only phoneme omission and substitution are considered as errors.
- Process Density Index (PDI)^(29)^: calculated from the speech samples of the ABFW Phonology test, represents the average of phonological processes used in the words of the speech sample. To obtain this measure, the total number of occurrences of phonological processes is calculated and divided by the number of words analyzed.
  • Speech Sound Stimulability Test (TESF, in Portuguese)^(30)^ for sounds absent from the phonetic inventory^(24)^. This is a word imitation task containing the target sound combined with the seven BP oral vowels. Sensory cues (articulatory, visual, and tactile) are used for this imitation. A target sound was considered stimulable when the child was able to produce it correctly in at least 10% of the stimuli offered.

The A1-B assessment was carried out 30 days after A1-A, when the children were again subjected to the SI, TSF-A, ABFW Phonology test to analyze phonological processes with more than 25% occurrence, calculate PCC, PCC-R, and PDI, and TESF.

After the multiple baseline assessments, the subjects were electronically randomized into two groups, using the random function of Microsoft Excel^®^: G1, which received the SSD intervention based on the adapted Cycles approach, through 12 synchronous video call sessions with the researcher; and G2, whose SSD intervention was similar, but carried out through synchronous meetings with the researcher in six of the 12 meetings, while the other six meetings were conducted by the caregiver, previously trained by the researcher.

### Post-intervention reassessments (A2-A and A2-B)

Post-SSD intervention assessments were performed 7 (A2-A) and approximately 30 to 40 days (A2-B) after the last intervention session to analyze the changes that the intervention promoted in the child's natural language development. The phonology imitation and naming tests of the ABFW Child Language Test^(24)^ were applied, calculating the number of phonological processes occurring more than 25%, PCC, PCC-R, and PDI.

### Interview on the use of digital resources

During the first meeting, an interview was conducted (Appendix A) regarding the familiarity of both the family and the child with the use of digital resources connected to the Internet, as well as technical information about the devices that the family had available for conducting online SSD-intervention sessions. Although they were instructed to use electronic devices with larger screens (such as computers, laptops, or tablets) so that the child could better view the images used in serious games, there was no control over the resources available to the family, nor the means of connecting to the Internet.

### Parents' perceptions of daily speech training

At the end of each intervention session, an email or text message via the WhatsApp instant messaging application was sent to the caregiver responsible for the daily training, containing instructions on how to perform the training and guidance on completing the digital form on the last day of training before the next session. The questions were open-ended, closed-ended, and some used the Likert scale and addressed the child's interest in training, training time, and understanding of the exercise, and the caregiver’s need for help to understand it, and perception of the child's speech.

### Intervention

The Speech Intervention Plan for Telepractice (Pifate, in Portuguese) for children with SSD is based on the adapted Cycles approach^(8)^. In each cycle, one of the most frequent phonological processes was selected, and for each of them, two stimulable target sounds were selected according to the TESF^(30)^, totaling four sounds.

To learn the target sound, different skills were addressed through playful activities during three sessions. All sessions began and ended with auditory stimulation through auditory bombardment. In session 1, activities involving the articulatory reinforcement of the target sound were carried out. In sessions 2 and 3, activities for auditory discrimination and phonological awareness were carried out, always with opportunities for the child to practice the production of the target sound. The approach to target sound 2 of phonological process 1, which had been addressed since session 1, began in session 4, repeating in sessions 4, 5, and 6 the same objectives of sessions 1, 2, and 3. The approach to phonological process 2 began in session 7, with the first target sound with its characteristics, and followed the same detailed structure of sessions 1, 2, and 3. The second target sound of phonological process 2 began in session 10, repeating the approaches detailed above, also in sessions 11 and 12. Hence, sessions 1, 4, 7, and 10; 2, 5, 8, and 11; and 3, 6, 9, and 12 have similar structures (Appendix B).

Each session lasted 50 minutes, and its management, as detailed in Chart 1, allowed for the creation and strengthening of the bond with the child, covering all the skills to be addressed, and guiding the parents at the end of each teleconsultation.

**Chart 1:** Organization of the objectives and therapeutic strategies of Pifate sessions

**Sessions**	**Objectives**	**Duration (m)**	**Strategies**
1, 4, 7, 10	Establish bond	5	Brief open conversation with the child
	Auditory stimulation	3	Auditory bombardment
	Presenting the target sound	7	TESF visual and phonetic feedback
	Articulatory reinforcement	10	Strategy 1: predefined WordWall^®^
		12	Strategy 2: The child chooses in Pink Cat Games^®^
	Auditory stimulation	3	Auditory bombardment
	Caregiver guidance	10	Session observations and guidance on training
2, 5, 8, 11	Establish bond	5	Brief open conversation with the child
	Auditory stimulation	3	Auditory bombardment
	Presenting minimal pairs	10 10	WordWall® game guiding the child to classify the minimal pairs.
	Auditory discrimination	10	Strategy 1: predefined WordWall®
			Strategy 2: The child chooses in Pink Cat Games®
	Auditory stimulation	3	Auditory bombardment
	Caregiver guidance	10	Session observations and guidance on training
3, 6, 9, 12	Establish bond	5	Brief open conversation with the child
	Auditory stimulation	3	Auditory bombardment
	Strengthening the phonological rule	12	Strategy 1: predefined WordWall®
		12	Strategy 2: The child chooses in Pink Cat Games®
	Auditory stimulation	3	Auditory bombardment
	Caregiver guidance	10	Session observations and guidance on training

All sessions were conducted via video call using Google Meet, recorded, and saved to ensure data security. The SLP was in a private, well-lit environment with direct light on her face, facing the camera of a laptop with a 1.6 GHz Intel Core i5 Dual-Core processor (MacBook), using Blackwire 3220 (Plantronics) headphones with a unidirectional microphone, connected to the Internet with download and upload speeds above 570 Mbps to ensure better quality of image and sound transmission. Parents were instructed to sit the child with supported feet in a quiet and well-lit environment, preferably using a tablet or computer (since they have larger screens) and headphones. The playful resources used during the sessions and sent for daily training were developed on the WordWall® and Pink Cat Games^®^ platforms.

### Parental guidance for G1 and G2

At the end of each session, the researcher sent instructions via email or WhatsApp (a well-known instant messaging application), according to the caregiver's preference. The messages regarding the training to be performed at home after the session with the SLP were similar for both groups, as specified in Chart 2.

**Chart 2:** Parental guidelines for daily home training (G1 and G2) and for the parenting session (G2)

**GUIDANCE MESSAGE CONTENT**		**DAILY TRAINING G1 AND G2**	**PARENTING SESSION G2**
**Managing the setting:**	Control noise in the area.	X	X
	Well-lit room	X	X
**Ergonomics:**	Child sitting, with feet supported, and electronic device on the table	X	X
**Equipment:**	Choose an electronic device with the largest possible screen.	X	X
	Use headphones during auditory bombardment		X
**Training:**	Train daily	X	
	Learn more about the target sound of the training (material sent to parents)	X	
	Learn more about the objective of training and each game	X	
	Find out the names of the figures used in each game (material sent to parents)	X	
	Read/watch the tutorial on how to play each shared game	X	
	Send the video recording of a section of the training to the speech therapist	X	
	Send questions, if you have any, to the speech therapist	X	
**Managing time**	Control the session duration (30 to 40 min)		X
	Conduct the session calmly, without rushing the child.		X
	Record videos of parts of the session		X
**Articulatory pattern model**	Stand in front of the child to give the articulatory model of the target sound		X
	Speak slowly and emphasize the target sound		X
	Use the tactile cues taught		X
**Intervention session**	Auditory stimulation: perform auditory bombardment using recorded audio sent via email		X
	Presenting minimal pairs: name the figures in each pair (use the answer key) with the child		X
	Open the link and access the games that will be the strategies for what we want to stimulate today. Read the written tutorial and/or watch the video on how to access and play		X
	End the session with the same auditory bombardment as at the beginning of the session.		X
	Record video clips of the session and upload		X
	Send your questions about the session		X
	Wait for the next email with the daily training to be carried out until the next session.		X
**Post-training:**	Send videos of the training you did before the next session	X	
	Answer the form saying what you thought of the interest, ease/difficulty in the child's execution of the task, and what you thought of the result of the child's speech.	X	

Only after their feedback were the parents in G2 sent instructions and activities for the session that they would complete at home without the synchronous participation of the SLP. After the session, the caregiver sent a new video sample of the session to the SLP, who then prepared the training email based on the video analysis.

### Guidance for caregiver-led intervention in G2

Sessions 1, 4, 6, 7, 10, and 12 were synchronous with the SLP, and sessions 2, 3, 5, 8, 9, and 11 were conducted by the previously instructed caregiver.

In session 1, the SLP performed the intervention with the child under the observation of the caregiver. At the end of the session, approximately 20 minutes were reserved for answering questions and training in the application of auditory bombardment, articulatory training, correcting incorrect articulatory production, and positive reinforcement of correct production (Appendix C).

The message of delivery and guidance regarding daily training at home was similar to the message delivered to G1. After families sent videos of excerpts of the training sessions performed with the child at home, together with the online interview describing their perception of the daily training performed, the message regarding session 2 was sent, now containing the session strategies for parental guidance, detailing how the session should be performed at home and covering the topics presented in Chart 2.

### Data analysis

Descriptive analysis was used to characterize the sample. The assessment of the difference between the groups regarding age was performed using Fisher's exact test, and regarding sex, using the Mann-Whitney test, with a significance level of 0.05.

The responses from the initial interview regarding the reason for the child's use of digital resources and the time of use were compared between the groups using Fisher's exact test and Mann-Whitney test, respectively, with a significance level of 5%.

To determine the severity of SSD, a multiple baseline analysis (Assessments A1-A and A1-B) was performed regarding the PCC, PCC-R, PDI, and the occurrence of phonological processes. The comparison between individuals and between groups was made using the Mann-Whitney test, with a significance value of 0.05.

The effectiveness of the intervention was analyzed based on the effect size, calculated considering the individual responses of each variable: number of different phonological processes with more than 25% occurrence in the imitation test; number of different phonological processes with more than 25% occurrence in the naming test; PCC and PCC-R obtained in the imitation test; PCC and PCC-R obtained in the naming test; PDI obtained in the imitation test; and PDI obtained in the naming test.

Thus, the effect size of each variable was obtained by the difference between the mean of the result between the multiple baseline (pre-intervention) and the mean of the result between post-intervention assessments, divided by the variability (standard deviation) between the pre-intervention multiple baseline results (A1-A and A1-B). When the post-intervention values were lower than the pre-intervention values, the result was indicated with a negative sign (-), for example, in PDI and Average Productivity of Phonological Processes. The effect size can be classified as small, medium, and large^(26)^.

The variables used to assess parents' perception of speech training performed at home with the child, which the parents completed after the training performed at home, were compared between the groups using the Mann-Whitney test with a significance level of 0.05.

## RESULTS

G1 had five male children, aged 5:0 to 7:2 years, with SSD severity ranging from mild to moderately severe. G2 had three male children and two female children, aged 5:2 to 7:7 years, with SSD severity ranging from mild to slightly moderate, with no statistically significant difference between the groups (sex p-value: 0,444; age p-value: 0,344; SSD severity p-value: >0,999).

### Comparison between G1 and G2 in A1

The comparison between the two groups in A1 did not show a statistically significant difference regarding the number of phonological processes occurring in the imitation (p-value = 0.114) and naming (p-value = 0.662) tests. The same occurred for the PCC (p-value = 0.421), PCC-R (p-value = 0.421), and PDI (p-value = 0.402) in the imitation and naming tests (PCC: p-value = 0.753; PCC-R: p-value = 0.833; PDI: p-value = 0.675).

### Family digital literacy

All families had Internet access via Wi-Fi. All children had controlled access to the Internet to watch videos and play digital games. Although there was no statistically significant difference between the groups regarding the use of electronic devices connected to the Internet, a longer daily time of device use was observed in G1 (41 to 60 minutes, while G2 = 21 to 30 minutes; p-value = 0.594).

### Description of each subject's performance in the study variables

The effect size was calculated^(26)^ considering the number of phonological processes with an occurrence greater than 25% in the imitation and naming tests; PCC and PCC-R in the imitation and naming tests; PDI in the imitation and naming tests.

**Figure 1:**
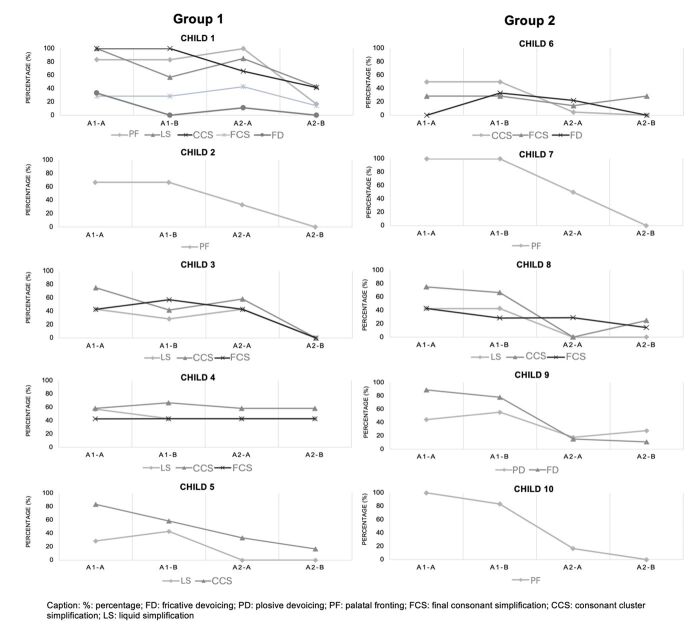
Percentage of occurrence of each phonological process in the imitation test in assessments A1-A, A1-B, A2-A, and A2-B per child

Figure 1 shows the occurrence of each phonological process in the imitation test in assessments A1-A, A1-B, A2-A, and A2-B per child. Table 1 shows the average results for each process before and after the intervention and the effect size for each process per child.

**Table 1:** Average occurrence of phonological processes in the pre- and post-intervention imitation test and effect size for each phonological process per child

	**Average occurrence of phonological process pre-intervention**	**Average occurrence of phonological process post-intervention**	**Effect size**	**Mean effect size**	**Effect Size Classification**
*Child 1*					
PF	88.88%	8.34%	-8.361	-3.167	Medium
LS	80.71%	35.71%	-2.069		
CCS	88.67%	42.26%	-2.364		
FCS	33.33%	15.48%	-2.166		
FD	14.81%	0.00%	-0.873		
*Child 2*					
PF	66.67%	16.67%	-1.191	-1.191	Small
*Child 3*					
LS	38.09%	0.00%	-4.620	-4.630	Medium
CCS	58.33%	0.00%	-3.499		
FCS	47.61%	0.00%	-5.771		
*Child 4*					
LS	49.98%	42.86%	-0.707	-0.234	Small
CCS	62.49%	58.33%	-0.702		
FCS	42.86%	42.86%	0.707		
*Child 5*					
LS	35.72%	0.00%	-3.535	-3.064	Medium
CCS	70.83%	25.00%	-2.593		
*Child 6*					
LS	50.00%	2.33%	-2.073	-0.873	Small
CCS	28.57%	21.43%	-0.311		
FCS	16.67%	11.11%	-0.236		
*Child 7*					
PF	100.00%	25.00%	-5.357	-5.357	Medium
*Child 8*					
LS	42.86%	0.00%	-5.364	-5.554	Medium
CCS	70.84%	12.50%	-9.904		
FCS	35.71%	21.64%	-1.393		
*Child 9*					
PD	50.00%	22.82%	-3.459	-6.186	Medium
FD	83.33%	13.25%	-8.913		
*Child 10*					
PF	91.67%	8.34%	-7.069	-7.069	Large

Caption: %: percentage; FD: fricative devoicing; PD: plosive devoicing; PF: palatal fronting; FCS: final consonant simplification; CCS: consonant cluster simplification; LS: liquid simplification

The average effect size of all phonological processes produced per child was calculated to compare the effect size between groups, considering the variability of processes observed in each child. The averages per child were compared between groups using the Mann-Whitney test. A tendency towards statistical significance was observed, with G2 presenting a larger effect size than G1 (Table 2).

**Table 2:** Comparison between G1 and G2 regarding the average effect size in the analysis of the number of different phonological processes with occurrence > 25% in the imitation test

**Group**	**Minimum**	**Maximum**	**Median**	**Mean**	**Standard deviation**	**Mann-Whitney test**	**p-value**
Group 1	-4.630	-0.234	-3.064	-2.457	1.742	21.000	0.095#
Group 2	-7.069	-0.873	-5.554	-5.008	2.406		

# p-value with tendency towards statistical significance

### Number of Different Phonological Processes Occurring at More Than 25% in the Naming Test in Multiple Baseline and Reassessments

**Figure 2:**
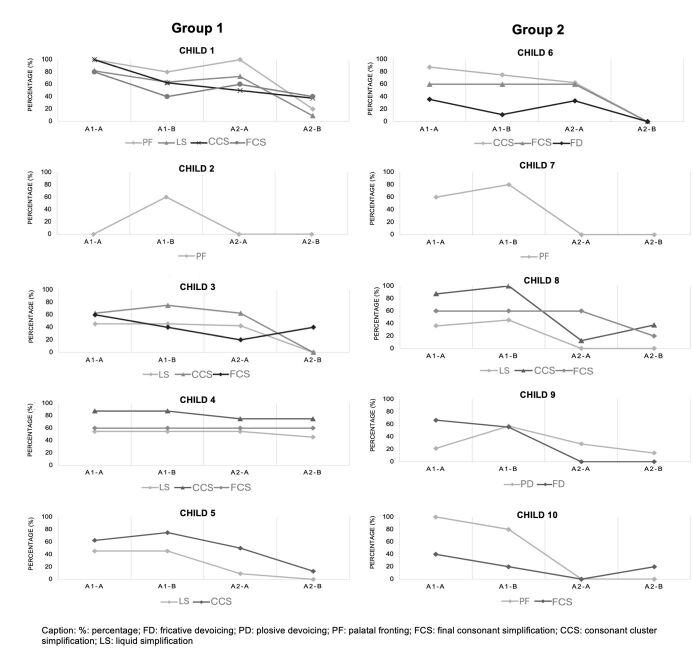
Percentage of occurrence of each phonological process in the naming test per child in assessments A1-A, A1-B, A2-A, and A2-B

Figure 2 shows the occurrence of each phonological process in the naming test in each of the assessments A1-A, A1-B, A2-A, and A2-B per child. Table 3 shows the average results per process before and after the intervention and the effect size for each process per child.

**Table 3:** Average occurrence of phonological processes in the pre- and post-intervention naming test and effect size for each phonological process per child

	**Average occurrence of phonological process pre-intervention**	**Average occurrence of phonological process post-intervention**	**Effect Size**	**Mean Effect Size**	**Effect Size Classification**
*Child 1*					
PF	93.33%	10.00%	-7.217	-4.063	Medium
LS	72.72%	13.55%	-6.513		
CCS	70.83%	31.25%	-1.521		
FCS	60.00%	40.00%	-1.000		
*Child 2*					
PF	30.00%	0.00%	-0.707	-0.707	Small
*Child 3*					
LS	44.45%	0.00%	-25.663	-11.967	Large
CCS	66.67%	0.00%	-9.238		
FCS	40.00%	20.00%	-1.000		
*Child 4*					
LS	54.54%	50.00%	-0.853	-1.066	Small
CCS	87.50%	75.00%	-2.345		
FCS	60.00%	60.00%	0.000		
*Child 5*					
LS	45.45%	4.55%	-4.633	-4.423	Medium
CCS	68.75%	31.50%	-4.214		
*Child 6*					
LS	81.25%	31.25%	-5.657	-0.273	Small
CCS	60.00%	30.00%	-2.308		
FCS	23.41%	16.67%	-0.388		
*Child 7*					
PF	70.00%	0.00%	-4.950	-4.950	Medium
*Child 8*					
LS	40.91%	0.00%	-6.364	-5.588	Medium
CCS	93.75%	25.00%	-7.778		
FCS	60.00%	40.00%	-2.621		
*Child 9*					
PD	39.28%	21.29%	-0.712	-4.242	Medium
FD	61.11%	0.00%	-7.772		
*Child 10*					
PF	90.00%	0.00%	-6.364	-3.889	Medium
FCS	30.00%	10.00%	-1.414		

Caption: %: percentage; FD: fricative devoicing; PD: plosive devoicing; PF: palatal fronting; FCS: final consonant simplification; CCS: consonant cluster simplification; LS: liquid simplification

The mean effect size of all phonological processes produced by the child was calculated to compare the effect size between the groups, considering the variability of processes observed in each child. Also, the means per child were compared between the groups using the Mann-Whitney test. No significant difference was observed between the groups, suggesting a similarity in the efficacy of the treatment regardless of the therapeutic protocol (Table 4).

**Table 4:** Comparison between groups regarding the number of different phonological processes with occurrence > 25% in the naming test

**Group**	**Minimum**	**Maximum**	**Median**	**Mean**	**Standard deviation**	**Mann-Whitney test**	**p-value**
Group 1	-11.967	-0.707	-4.063	-4.445	4.531	13.000	>0.999
Group 2	-5.588	-0.273	-4.242	-3.788	2.071		

### PCC and PCC-R in the imitation and naming tests in multiple baseline and reassessments

**Figure 3:**
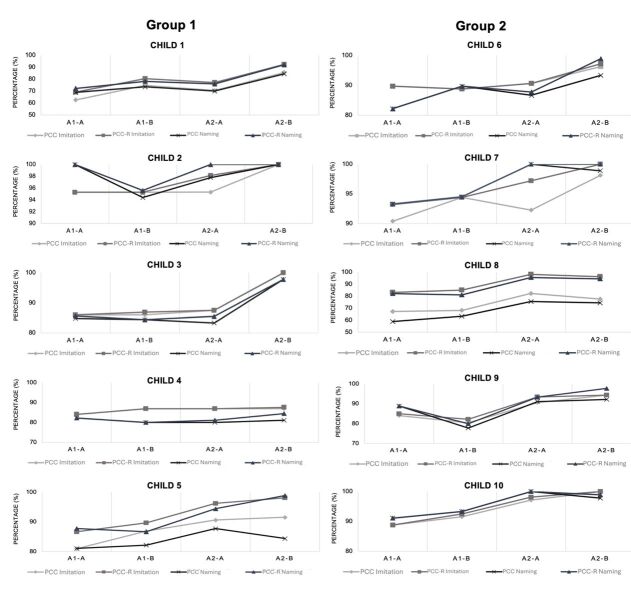
PCC and PCC-R obtained in the imitation and naming tests per child in the A1-A, A1-B, A2-A, and A2-B assessments

Figure 3 shows the PCC and PCC-R obtained in both the imitation and naming tests in each of the assessments A1-A, A1-B, A2-A, and A2-B per child.

The classification of the effect size in relation to the mean PCC and PCC-R in the imitation test before and after the intervention ranged from small to large for both G1 and G2 subjects. When comparing the effect size between the groups, no significant difference was observed, suggesting a similarity in the efficacy of the treatment regardless of the therapeutic protocol (Table 5).

**Table 5:** Comparison of the effect size between G1 and G2 for PCC and PCC-R in the imitation and naming test

	**Minimum**	**Maximum**	**Median**	**Mean**	**Standard deviation**	**Mann-Whitney test**	**p-value**
*PCC Imitation*							
Group 1	0.667	15.588	1.717	4.331	6.362	6.000	0.222
Group 2	0.988	19.108	4.235	6.973	7.071		
*PCC-R Imitation*							
Group 1	0.881	17.484	3.085	5.351	6.927	6.000	0.222
Group 2	3.216	9.710	5.657	6.224	2.436		
*PCC Naming*						13.000	>0.999
Group 1	-0.350	18.968	5.734	6.153	7.738		
Group 2	0.764	6.541	4.300	3.426	2.462		
*PCC-R Naming*							
Group 1	0.707	20.626	5.300	7.963	8.436	12.000	>0.999
Group 2	1.393	17.163	4.654	6.433	6.444		

### PDI in the imitation and naming tests in multiple baseline and reassessments

**Figure 4:**
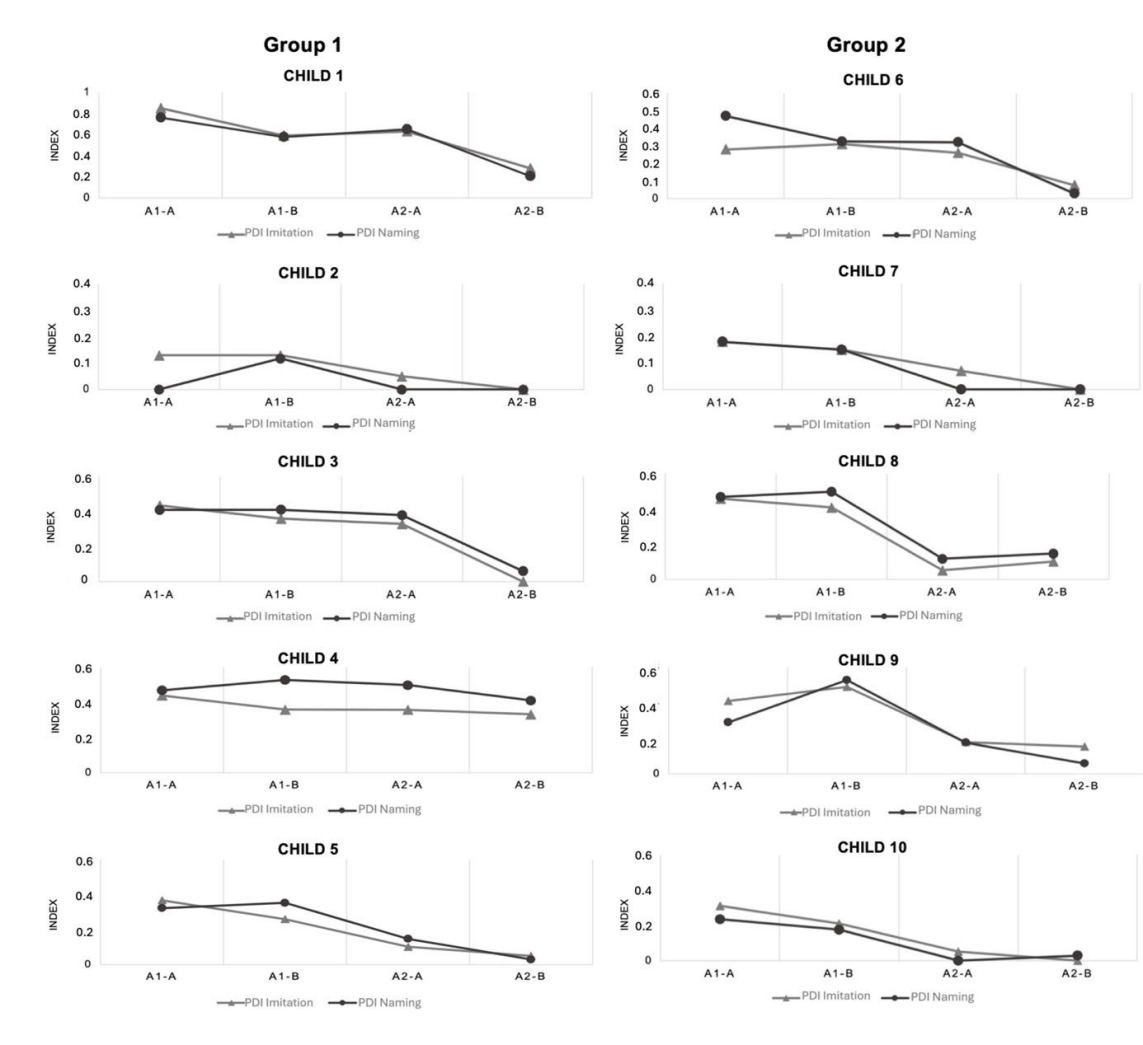
PDI obtained in the imitation and naming tests per child in the A1-A, A1-B, A2-A, and A2-B assessments

Figure 4 shows the PDI in both the imitation and naming tests in each of the assessments A1-A, A1-B, A2-A, and A2-B per child.

As in the classification of the effect size in relation to the mean PCC and PCC-R, the comparison of the effect size of PDI in the imitation test before and after the intervention varied from small to large for both the G1 and G2 subjects, showing no statistically significant difference.

### Parents’ perceptions of speech training at home with their children

The time of child interest in each of the two training activities was on average 11 to 15 minutes in both groups, with no significant difference between the groups according to the Mann-Whitney test (p-value > 0.999).

**Figure 5:**
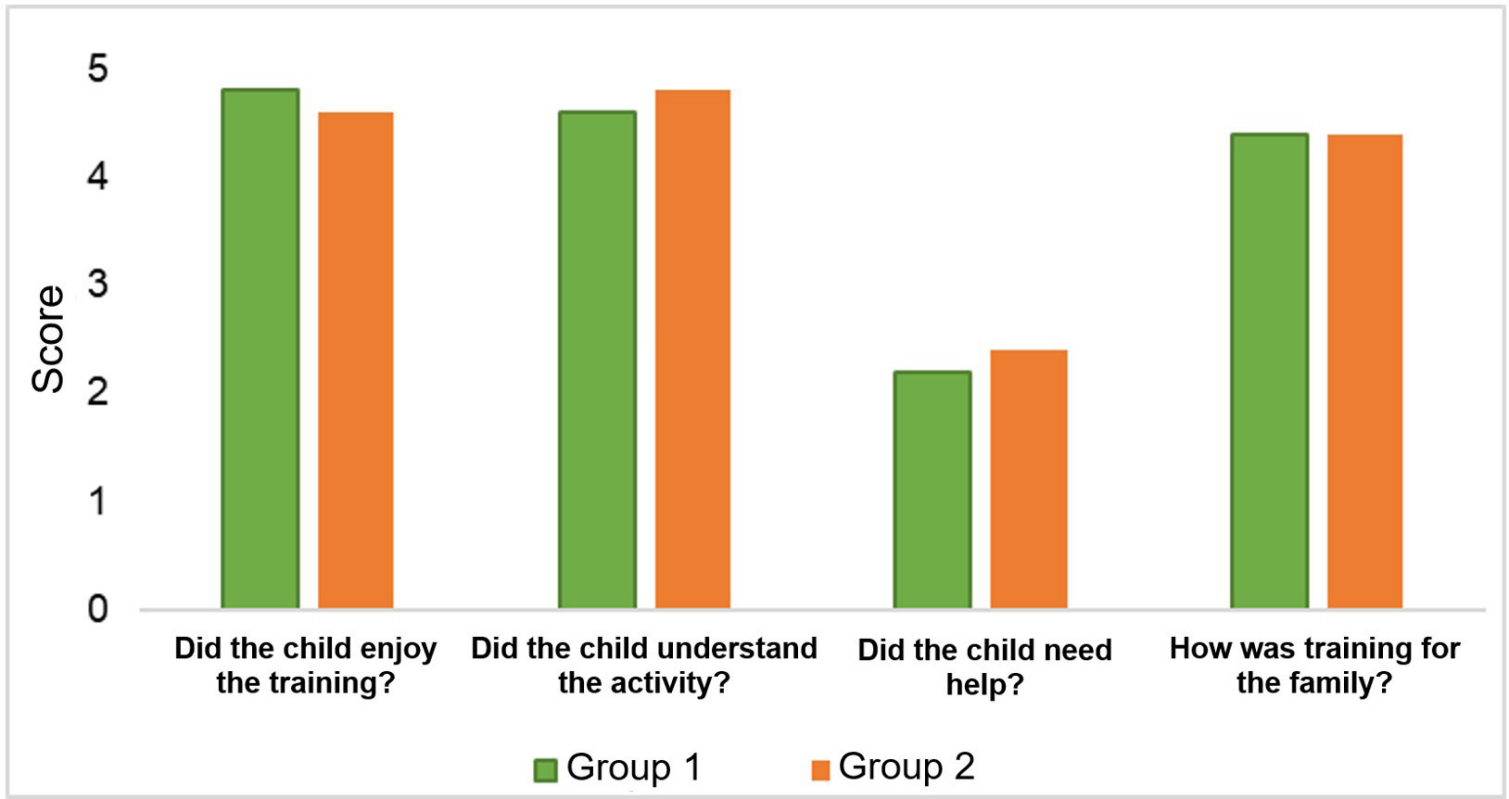
Average responses regarding families' perception of daily training

According to the assessment of parents in both groups, the children showed great interest in the activities proposed for training at home, indicating an average response of 4 points on a 5-point Likert scale, with no significant difference between the groups according to the Mann-Whitney test (p-value = 0.600) (Figure 5).

The children were instructed by their parents on how to practice the stimulated target sound at home. According to their parents, they found it easy to understand these instructions, achieving an average of 4 points in this question in both groups, requiring little support to carry out the training, with an average of 2 points for both groups. The comparison of the groups for both questions showed no significant difference between them by the Mann-Whitney test (p-value = 0.600 and p-value = 0.911) (Figure 5).

When asked about how it was to carry out speech training with the child, the average response was 4 points in both groups, with no significant difference between them according to the Mann-Whitney test (p-value > 0.999) (Figure 5).

## DISCUSSION

Applying telepractice in SSD intervention with children is a solution that enables access to treatment even in challenging situations, such as great distances between the family and the professional, difficulty in moving, and in critical situations^(1,9,14)^, as occurred during the COVID-19 pandemic, enabling professional monitoring even during months of social isolation^(31)^.

The use of serious digital games focused on speech learning and platforms that offer editing and customization features for such games^(10,32)^ further expanded the resources for online clinical practice.

Researchers aimed to compare the effectiveness of playful strategies with physical material and playful strategies with digital serious games used in in-person sessions in the intervention of children with SSD^(33,34)^. They found that both groups improved in speech intelligibility after the intervention. Thus, they observed that using digital games as therapeutic resources is as effective as the traditional approach with physical resources on a table in the treatment of children with idiopathic SSD.

The results of the present study showed that Pifate was effective for all subjects, with effect sizes ranging from small to large, reducing the occurrence of phonological processes (indicated by the comparison of phonological process productivity) and improving speech intelligibility (indicated by the comparison of PCC, PCC-R, and PDI) for both intervention groups, with no statistically significant difference between them. The variations in effect size between subjects may be related to the different intrinsic characteristics of each subject. Researchers^(35)^ studied the effect of applying multiple oppositions in the treatment of SSD in children and analyzed parental participation in the delivery of the intervention. Their findings were also positive, with variation in effect size between subjects, further indicating that properly trained parents can adequately conduct their children's speech-language intervention sessions.

Keeping parents engaged throughout the process was also a therapeutic goal, regardless of whether they were part of G1 or G2. In addition to asking parents to attend all sessions with the SLP, thus ensuring learning and greater aptitude for applying the training with the child, guidance materials were also prepared for training in all sessions, with simple texts and examples of what they were asked to apply with the children. According to a recent study^(36)^, guided and informative communication between the SLP and the parents ensures greater engagement of the parents in carrying out the daily training, and it is often necessary to change the guidance strategies according to the characteristics of each caregiver.

Researchers^(37)^ reflect that there is still a culture of in-person healthcare, and it is important to consider training for both professionals and children and parents to make better use of the services offered through telepractice. The strategies applied by parents were monitored by sharing videos of part of the training and sessions carried out at home with the child, as well as by filling out an online form with questions encouraging a critical analysis of the training at home. Thus, the SLP could guide the parents, adjust details, and provide words of praise as an incentive for them to continue or improve in the training in the following sessions.

Knowing the digital literacy profile was important to ensure that large differences in this aspect between the subjects did not interfere with the results. G1 and G2 caregivers and children were not statistically significantly different in literacy profile. However, the children in G1 used an electronic device for longer during the day. The socioeconomic profile and education level of the parents were not investigated in this study, and it was not possible to consider them in the hypotheses throughout the discussion.

Parental engagement was essential for the children’s results in both groups. The effect size^(26)^ of the intervention on the research subjects ranged from small to large, with the difference between the groups tending toward statistical significance, indicating a larger effect size in G2. Even though it is only a trend, which could be confirmed or not if the study continued with the recruitment of more subjects in each group, it is interesting to point out that the group with fewer synchronous sessions with the SLP tended to have a larger effect size than the group that carried out all synchronous sessions with the professional. One hypothesis to explain this tendency towards statistical difference is that when parents are more involved in the therapeutic process, they may perform better in daily training with the child, as well as inserting correction and speech production strategies in the child's daily activities, generating greater assimilation and automation of the target sound by the child^(20)^.

When analyzing^(38)^ the parents' perception of the experience with telepractice in the treatment of their children, it was observed that the parents valued the active role they played in their children's therapy, despite the practical and emotional challenges associated with telepractice. It is also important to measure the level of complexity of the speech training requested from the parents and deliver activities that the parents consider easy to carry out with the children, as these are more acceptable to them.

Although the dose and frequency of stimuli offered by the SLP were similar for both groups, it can be hypothesized that families in G2 spontaneously offered daily training and session strategies at a higher frequency, thus increasing the cumulative intensity of the intervention^(39)^ and the tendency towards a greater effect size of Pifate in this group.

However, it is important to highlight the limitations of the study due to the small sample size, which made statistical analysis difficult when comparing the groups. Therefore, the study must continue with more subjects per group to confirm the trend towards a statistically significant difference with better results in group 2 than in group 1.

## CONCLUSION

This study demonstrated the effectiveness of Pifate, showing that the adapted Cycles intervention approach can be applied through telepractice.

Both groups performed similarly, suggesting that synchronous sessions with the SLP through telepractice, as well as with parents previously guided by the SLP, improved the child's speech. The guidance and training of parents by the SLP through telepractice were essential to provide good engagement in the sessions and daily tasks at home.

## References

[1] ASHA: American Speech-Language-Hearing Association (2024). Articulation and phonology.

[2] Eadie P, Morgan A, Ukoumunne OC, Ttofari Eecen K, Wake M, Reilly S (2015). Speech sound disorder at 4 years: prevalence, comorbidities, and predictors in a community cohort of children. Dev Med Child Neurol.

[3] Ceron MI, Gubiani MB, Oliveira CR, Gubiani MB, Keske-Soares M (2017). Ocorrência do desvio fonológico e de processos fonológicos em aquisição fonológica típica e atípica. CoDAS.

[4] Ingram D (1976). Phonological disability in children..

[5] Wertzner HF, Amaro L, Galea DE (2007). Phonological performance measured by speech severity indices compared with correlated factors. Sao Paulo Med J.

[6] Hodson BW (2006). Identifying phonological patterns and projecting remediation cycles: expediting intelligibility gains of a 7 year old Australian child. Adv Speech Lang Pathol.

[7] Hodson BW (2011). Enhancing phonological patterns of young children with highly unintelligible speech. ASHA Lead.

[8] Wertzner HF, Pagan-Neves LO, Pro-Fono (2015). Planos terapêuticos fonoaudiológicos (PTFs)..

[9] Lopes A, Nielsen C, Ferrari D, Campos P, Ramos SM (2020). Diretrizes de boas práticas em Telefonoaudiologia.

[10] Attwell GA, Bennin KE, Tekinerdogan B (2023). Reference architecture design for computer-based speech therapy systems. Comput Speech Lang.

[11] Breuer J, Bente G (2010). Why so serious? On the relation of serious games and learning. Eludamos (Gött).

[12] McLeod S, Verdon S, Tran VH, Margetson K, Wang C (2022). SuperSpeech: multilingual speech and language maintenance intervention for Vietnamese-Australian children and families via telepractice. Lang Speech Hear Serv Sch.

[13] Saeedi S, Bouraghi H, Seifpanahi M-S, Ghazisaeedi M (2022). Application of digital games for speech therapy in children: a systematic review of features and challenges. J Healthc Eng.

[14] Furlong L, Erickson S, Morris ME (2017). Computer-based speech therapy for childhood speech sound disorders. J Commun Disord.

[15] Grogan-Johnson S, Schmidt AM, Schenker J, Alvares R, Rowan LE, Taylor J (2013). A comparison of speech sound intervention delivered by telepractice and side-by-side service delivery models. Comm Disord Q.

[16] Coufal K, Parham D, Jakubowitz M, Howell C, Reyes J (2018). Comparing traditional service delivery and telepractice for speech sound production using a functional outcome measure. Am J Speech Lang Pathol.

[17] Lee SAS (2018). The treatment efficacy of multiple opposition phonological approach via telepractice for two children with severe phonological disorders in rural areas of West Texas in the USA. Child Lang Teach Ther.

[18] Gürsoy MD, Tığrak TK, Köse A (2022). Telepractice with preschool children: speech-language pathologists’ perspectives in Turkey. Int J Telerehabil.

[19] Sugden E, Baker E, Munro N, Williams AL (2016). Involvement of parents in intervention for childhood speech sound disorders: a review of the evidence. Int J Lang Commun Disord.

[20] Law J, Roulstone S, Lindsay G (2015). Integrating external evidence of intervention effectiveness with both practice and the parent perspective: development of ‘What Works’ for speech, language, and communication needs. Dev Med Child Neurol.

[21] McFaul H, Mulgrew L, Smyth J, Titterington J (2022). Applying evidence to practice by increasing intensity of intervention for children with severe speech sound disorder: a quality improvement project. BMJ Open Qual.

[22] Shriberg LD, Austin D, Lewis BA, McSweeny JL, Wilson DL (1997). The percentage of consonants correct (PCC) metric: extensions and reliability data. J Speech Lang Hear Res.

[23] Barrozo TF, Pagan-Neves LO, Silva JP, Wertzner HF (2017). Sensibilidade e especificidade da Porcentagem de Consoantes Corretas Revisada na identificação do transtorno fonológico. CoDAS.

[24] Wertzner HF, Andrade CRF, Befi-Lopes DM, Fernandes FDM, Wertzner HF (2004). ABFW: teste de linguagem infantil nas áreas de fonologia, vocabulário, fluência e pragmática..

[25] Castro MM, Wertzner HF (2011). Speech inconsistency index in brazilian portuguese-speaking children. Folia Phoniatr Logop.

[26] Gierut JA, Morrisette ML, Dickinson SL (2015). Effect size for single-subject design in phonological treatment. J Speech Lang Hear Res.

[27] Herrero SF (2007). Desempenho de crianças com transtorno fonológico no teste de sensibilidade fonológica e de leitura e escrita.

[28] Shriberg LD, Kwiatkowski J (1982). Phonological disorders III: a procedure for assessing severity of involvement. J Speech Hear Disord.

[29] Edwards ML (1992). Phonological assessment and treatment in support of phonological processes. Lang Speech Hear Serv Sch.

[30] Castro MM, Pagan-Neves LO, Barrozo TF, Francisco DT, Wertzner HF (2022). TESF: teste de estimulabilidade dos sons da fala..

[31] McLeod S, Ballard KJ, Ahmed B, McGill N, Brown MI (2020). Supporting children with speech sound disorders during covid-19 restrictions: technological Solutions. Perspect ASHA Spec Interest Groups.

[32] McLeod S, Kelly G, Ahmed B, Ballard KJ (2023). Equitable access to speech practice for rural australian children using the SayBananas! mobile game. Int J Speech Lang Pathol.

[33] Jesus LMT, Martinez J, Santos J, Hall A, Joffe V (2019). Comparing traditional and tablet-based intervention for children with speech sound disorders: a randomized controlled trial. J Speech Lang Hear Res.

[34] Silva TF, Ribeiro GCF, Silva CEE, Assis MF, Dezani H, Berti LC (2023). Eficácia no uso de estratégia de gamificação na terapia fonológica. CoDAS.

[35] Sugden E, Munro N, Trivette CM, Baker E, Williams AL (2019). Parents’ experiences of completing home practice for speech sound disorders. J Early Interv.

[36] Tambyraja SR (2020). Facilitating parental involvement in speech therapy for children with speech sound disorders: a survey of speech-language pathologists’ practices, perspectives, and strategies. Am J Speech Lang Pathol.

[37] Nickbakht M, Meyer C, Scarinci N, Beswick R (2020). Exploring factors influencing the use of an eHealth intervention for families of children with hearing loss: an application of the COM-B model. Disabil Health J.

[38] Pozniak K, Rosenbaum P, Kwok EYL (2024). Tasks performed by parents to enable telepractice for children with communication disorders: an interview study with clinicians and parents. Disabil Rehabil.

[39] Warren SF, Fey ME, Yoder PJ (2007). Differential treatment intensity research: a missing link to creating optimally effective communication interventions. Ment Retard Dev Disabil Res Rev.

