# Supplementary material for: Efficacy of the adapted Cycles approach in telepractice speech-language pathology with a parental focus for children with speech sound disorders
Source: Codas. 2025 Aug 11;37(4):e20240216. doi: 10.1590/2317-1782/e20240216en (PMC12341760; doi:10.1590/2317-1782/e20240216en)
Supplement: Translation

# Translation: Eficácia do plano de intervenção da abordagem dos Ciclos adaptada por telefonoaudiologia com enfoque parental para crianças com transtornos dos sons da fala

**DOI:** 10.1590/2317-1782/e20240216pt

**Authors:** Daniela Aparecida Barbosa, Haydée Fiszbein Wertzner

**Affiliations:** 1 Departamento de Fisioterapia, Fonoaudiologia e Terapia Ocupacional, Faculdade de Medicina, Universidade de São Paulo – USP - São Paulo (SP), Brasil.

**Keywords:** Transtorno Fonológico, Teleconsulta, Telemonitoramento, Linguagem Infantil, Fonoterapia, Gamificação, Percepção de Fala, Pais

## Abstract

**Objetivo:**

Avaliar a eficácia da abordagem dos Ciclos adaptada à telefonoaudiologia e o engajamento familiar no tratamento de crianças com transtorno dos sons da fala (TSF).

**Método:**

Participaram 10 crianças, de 5:0 a 7:8 anos, com diagnóstico de TSF. Avaliou-se utilizando linha de base múltipla e duas avaliações pós-tratamento. A eficácia foi medida comparando pré e pós-intervenção para a porcentagem de consoante correta (PCC), porcentagem de consoante correta revisada (PCC-R), Process Density Index (PDI) e número de processos fonológicos com ocorrência >25%, nas provas de fonologia do teste ABFW, calculando-se o tamanho do efeito (TE) da intervenção. A percepção das famílias sobre os treinos foi obtida por entrevista e escala Likert. Os sujeitos foram distribuídos aleatoriamente em G1: realizou duas sessões online semanais com a fonoaudióloga, e G2: realizou metade das sessões conduzidas pelo cuidador treinado pela fonoaudióloga. Ambos receberam 12 sessões e realizaram treinos diários de fala.

**Resultados:**

Todos os participantes apresentaram aumento em PCC, PCC-R e redução em PDI e no número de processos fonológicos com ocorrência >25%, com TE variando de pequeno a grande. Houve tendência à significância estatística (TE G2>G1). Todos os pais relataram grande interesse das crianças pelas atividades, com boa experiência familiar (Likert=4) ao treinar a fala da criança usando recursos digitais.

**Conclusão:**

A eficácia da abordagem proposta variou de médio a alta. O desempenho dos grupos foi semelhante, com tendência à maior eficácia para o G2, focado na família. O treinamento dos pais resultou em bom engajamento nas sessões e treinos diários.

## INTRODUÇÃO

Os Transtornos dos Sons da Fala (TSF) são caracterizados por dificuldades na produção motora, percepção auditiva e/ou habilidades cognitivo-linguísticas, afetando a percepção e produção dos sons da fala^(1)^. Esses transtornos estão entre os mais comuns na população pediátrica, com prevalência estimada entre 2,3% e 24,6%^(1,2)^. No Brasil, a ocorrência pode variar entre 8,26% e 20,63%^(3)^.

Durante o desenvolvimento típico da linguagem, espera-se que as crianças, gradativamente, superem os processos fonológicos até aproximadamente os sete anos de idade, atingindo uma fala sem simplificações fonológicas^(4)^.

Os processos fonológicos, que caracterizam o TSF, envolvem padrões de erros que simplificam a regra fonológica da língua, sendo observados durante o desenvolvimento fonológico típico^(4)^. A manutenção de processos fonológicos além da idade esperada para sua eliminação, é o indicativo de possivelmente tratar-se de TSF^(5)^.

A abordagem dos Ciclos é fundamentada na interação dos processos cognitivo-linguístico, perceptivo e de produção de fala^(6,7)^. Os fundamentos da abordagem dos Ciclos consideram que a aquisição fonológica é um processo gradual; a aquisição dos sons da fala ocorre ouvindo os falantes; a associação de sensações cinestésicas e auditivas possibilita a automatização do som adquirido; a estimulação de novos sons da fala facilita sua produção correta; o envolvimento da criança em sua aquisição fonológica; a generalização das habilidades fonológicas para outros sons aprendidos espontaneamente; o uso da análise fonológica para iniciar o tratamento em um nível funcional para a criança^(6)^.

No Brasil, a abordagem dos Ciclos adaptada é aplicada às crianças com inventário fonético incompleto, com presença de processos fonológicos não esperados para a idade e com ocorrência superior a 25%, dificuldade na discriminação auditiva e produção de alguns sons da fala e dificuldades na consciência fonológica^(8)^.

Mais recentemente as diferentes formas de entrega da intervenção têm sido estudadas, como presencial e, por meio da telefonoaudiologia, virtual síncrona e assíncrona^(9,10)^.

### Intervenção no TSF por telefonoaudiologia

A telefonoaudiologia permitiu importante ganho na entrega do tratamento fonoaudiológico às crianças e adesão das famílias ao mesmo, por transpor as barreiras relacionadas à distância e deslocamento. Além disso, com o uso dos jogos digitais sérios^(11-13)^, que são jogos com o objetivo de ensinamento de algum conceito, é possível manter a criança engajada durante a teleconsulta e interessada em realizar os treinos diários inerentes ao tratamento^(13)^. Assim, a telefonoaudiologia utilizando jogos digitais sérios tem o potencial de fornecer intervenção em maior dose e intensidade em comparação com as intervenções tradicionais presenciais^(14)^.

Estudos^(15,16)^ já apontavam que tanto a intervenção presencial quanto a entregue por meio da telefonoaudiologia são eficazes, com a vantagem adicional da terapia online facilitar a prática intensiva e diária. Mesmo havendo risco de problemas da conexão com a internet e usabilidade dos recursos tecnológicos, a telefonoaudiologia para intervenções fonológicas e articulatórias tem eficácia validada para a população rural^(17)^ e escolar^(16)^.

Além dos recursos tecnológicos, a telefonoaudiologia é dependente também dos recursos humanos, sendo assim, a capacitação do fonoaudiólogo para utilização dos recursos digitais e manejo de possíveis problemas durante a teleconsulta, bem como o letramento digital da família com enfoque na comunicação e informação devem ser considerados antes da indicação desta modalidade de intervenção^(18)^.

É muito importante também compreender a telefonoaudiologia pelo olhar dos pais e cuidadores das crianças, uma vez que eles exercem fundamental papel no tratamento. O envolvimento dos pais na intervenção presencial das crianças com TSF ainda é pouco detalhado nos estudos^(19)^. É comum incluí-los na execução dos treinos diários e, para tal, realizar treinamento prévio por meio da observação da prática do fonoaudiólogo, realização de prática supervisionada pelo profissional e recebimento de instruções escritas. Este movimento vem se expandindo pois está clara a necessidade de descentralizar a intervenção fonoaudiológica do profissional e buscar uma parceria mais intensa com as famílias das crianças em tratamento^(20)^.

Estudo recente^(21)^, ao investigar os ganhos do aumento da frequência da dose de intervenção fonoaudiológica em dez crianças com TSF, verificou a variável PCC, bem como a percepção dos pais destas crianças tanto em relação à mudança da inteligibilidade de fala quanto em relação ao grau de satisfação com o tratamento. A elaboração do plano de implementação do aumento de frequência da intervenção contou com a colaboração de dois dos pais de crianças com TSF, o que pode ter contribuído para a assertividade da nova proposta para o maior engajamento das demais famílias participantes da pesquisa.

Diante da necessidade de maior conhecimento quanto ao uso da telefonoaudiologia nas intervenções dos TSF, o presente estudo teve por objetivo avaliar a eficácia da intervenção proposta e o engajamento da família na aplicação da abordagem dos Ciclos adaptada à telefonoaudiologia no tratamento de crianças com TSF.

## MÉTODO

Este estudo foi aprovado pelo Comitê de Ética em Pesquisa da Faculdade de Medicina na Universidade de São Paulo, sob o parecer número 3.340.790 e CAAE no. 10019019.8.0000.0065. Todos os pais e/ou responsáveis pelos sujeitos assinaram o Termo de Consentimento Livre e Esclarecido. Já os sujeitos foram previamente orientados sobre o processo de intervenção, utilizando para tal o Termo de Assentimento, onde a criança demonstrou seu aceite por meio de desenho ou assinatura.

### População do estudo

A população foi composta por dez díades cuidador (pai ou mãe)-criança de 5:0 a 7:11 anos, residentes nas cidades que compõem a Grande São Paulo.

Os critérios de inclusão foram ter idade entre 5:0 a 7:11 anos; sem tratamento prévio, ou encerrado/interrompido há pelo menos seis meses; ter como língua materna o Português Brasileiro (PB); sem queixas de alterações cognitivas, emocionais e/ou do neurodesenvolvimento; apresentar valor do índice de porcentagem de consoante correta revisada (PCC-R)^(22)^ inferior a 93,4%^(23)^ em pelo menos uma das provas de fonologia^(24)^ do Teste de Linguagem Infantil ABFW; ser consistente na prova de Inconsistência de Fala (IF)^(25)^; apresentar limiares auditivos dentro dos padrões de normalidade; integrar um núcleo familiar com letramento digital com enfoque em comunicação e aprendizado.

Os critérios de exclusão foram apresentar perda auditiva de qualquer grau, diagnosticada por meio de avaliação audiológica; outros comprometimentos de linguagem, cognitivo ou neurológico; não possuir meios de conexão com a internet; número de faltas nas sessões de tratamento superior a 3 alternadas.

### Coleta de dados

Foram realizadas avaliação inicial para o diagnóstico de TSF (A1-A), linha de base múltipla (A1-A e A1-B) para identificar a curva de desenvolvimento natural da criança^(26)^ e reavaliações (A2-A e A2-B) para verificar a influência da intervenção fonoaudiológica neste desenvolvimento. As amostras de fala da criança obtidas durante as avaliações foram registradas em vídeo com áudio, para posterior análise.

A percepção dos pais sobre o treino de fala realizado em casa junto à criança foi coletada ao longo da intervenção terapêutica, por meio de preenchimento de entrevista online com questões fechadas e abertas.

### Linhas de base múltiplas (A1-A e A1-B)

A A1-A foi realizada em 2 a 3 atendimentos, para evitar que o cansaço da criança interferisse em seu desempenho nos testes. Nela foram aplicadas a entrevista sobre uso de recursos digitais, avaliação audiológica, e uma bateria de testes, conforme listado a seguir:

- Teste de Sensibilidade Fonológica versão auditiva (TSF-A)^(27)^. Consiste em quatro provas que verificam o desempenho nas habilidades de aliteração igual, aliteração diferente, rima igual e rima diferente. Cada prova apresenta 15 itens, sendo os três primeiros para treino e 12 para aplicação e análise.
- Teste de Inconsistência de Fala (IF)^(25)^ para calcular o índice de inconsistência de fala. Composto por 25 figuras, nomeadas três vezes em sequências diferentes, e intercaladas por atividades distrativas. Cada uma das 25 palavras foi analisada e, se as três nomeações foram iguais, a produção da palavra foi considerada consistente.
- Provas de Fonologia (imitação de palavras e nomeação de figuras) do Teste de Linguagem Infantil ABFW^(24)^. A execução da prova foi registrada em áudio e vídeo e posteriormente realizada a transcrição fonética duas vezes pela pesquisadora a fim de garantir fidedignidade. Os processos fonológicos foram analisados de acordo com a orientação do teste Fonologia ABFW e aqueles com mais de 25% de ocorrência, em faixa etária superior à esperada para a superação foram considerados produtivos e alvos do tratamento. Foi calculado o número de diferentes processos fonológicos com ocorrência maior que 25%.
- Porcentagem de Consoante Correta (PCC)^(28)^: refere-se à porcentagem de consoantes produzidas corretamente em relação percentual ao total de consoantes contidas na amostra de fala analisada, sendo considerados como erros substituição, omissão e distorções de fonemas. Para este índice, os autores também classificam a gravidade como leve (acima de 85%), levemente moderada (65% a 85%), moderadamente grave (50% a 65%) ou grave (abaixo de 50%).
- Porcentagem de Consoante Correta – Revisada (PCC-R)^(22)^: difere do PCC o fato de serem considerados como erros somente omissão e substituição de fonemas.
- Process Density Index (PDI)^(29)^: calculado a partir das amostras de fala do teste de Fonologia ABFW, representa a média de processos fonológicos utilizados nas palavras da amostra de fala. Para obter essa medida, calcula-se o número total de ocorrências de processos fonológicos e divide pelo número de palavras analisadas.
  ● Teste de Estimulabilidade dos Sons da Fala (TESF)^(30)^ para os sons ausentes do inventário fonético^(24)^. Trata-se de uma tarefa de imitação de palavras contendo o som alvo combinado às sete vogais orais do PB. Para esta imitação são utilizadas pistas sensoriais (articulatória, visual e tátil). Um som alvo foi considerado estimulável quando a criança conseguia produzi-lo corretamente em pelo menos 10% dos estímulos oferecidos.

A avaliação A1-B foi realizada após trinta dias da A1-A, quando as crianças foram novamente submetidas à IF, TSF-A, Teste de Fonologia do ABFW para análise dos processos fonológicos com mais de 25% de ocorrência e cálculo de PCC, PCC-R e PDI e aplicação do TESF.

Após as avaliações das linhas de base múltiplas, os sujeitos foram randomizados eletronicamente, utilizando a função Aleatória do programa Microsoft Excel^®^ em dois grupos: G1 que recebeu a intervenção fonoaudiológica baseada na abordagem dos Ciclos adaptada, por meio de 12 sessões de videochamadas síncronas com a pesquisadora e G2 cuja intervenção fonoaudiológica foi semelhante, porém realizada por meio de encontros síncronos com a pesquisadora em seis dos 12 encontros e os outros seis encontros foram conduzidos pelo cuidador, previamente treinado pela pesquisadora.

### Reavaliações pós-intervenção (A2-A e A2-B)

As avaliações pós-intervenção fonoaudiológica foram realizadas sete (A2-A) e cerca de 30 a 40 dias (A2-B) após a última sessão de intervenção, a fim de analisar as mudanças que a intervenção promoveu no desenvolvimento natural da linguagem da criança. Foram aplicadas as provas de imitação e nomeação da Prova de Fonologia do Teste de Linguagem ABFW^(24)^ e calculadas o número de processos fonológicos com ocorrência maior que 25%, PCC, PCC-R e PDI.

### Entrevista sobre o uso de recursos digitais

Durante o primeiro encontro foi realizada uma entrevista (Apêndice A) quanto à familiaridade no uso dos recursos digitais conectados à internet tanto pela família quanto pela criança, bem como informações técnicas sobre os dispositivos que a família dispunha para a realização das sessões de intervenção fonoaudiológica online. Apesar de serem orientados a utilizar dispositivos eletrônicos com maior tela (como computador, laptop ou tablet), para que a criança tivesse melhor visualização das imagens utilizadas nos jogos sérios, não houve controle quanto aos recursos disponíveis pela família, assim como do meio de conexão com a internet.

### Percepção dos pais sobre o treino de fala diário

Ao final de cada sessão de intervenção foi enviado um e-mail ou mensagem de texto via aplicativo de mensagem instantânea *WhatsApp* ao cuidador responsável pelo treino diário, contendo as instruções sobre como realizar o referido treino e orientação para o preenchimento do formulário digital no último dia de treino antes da sessão seguinte. As questões eram abertas, fechadas e algumas utilizavam a escala Likert e abordavam o interesse da criança pelo treino, tempo de treino, compreensão do exercício pela criança, necessidade ou não de ajuda do cuidador para esta compreensão e a percepção da fala da criança pelo cuidador.

### Intervenção

O Plano de Intervenção da Fala por Telefonoaudiologia (Pifate) para crianças com TSF tem como base de apoio a proposta da abordagem dos Ciclos adaptada^(8)^. Em cada ciclo foi selecionado um dos processos fonológicos de maior ocorrência, e para cada um deles dois sons-alvo estimuláveis de acordo com o TESF^(30)^ totalizando então quatro sons.

Para o aprendizado do som alvo, durante três sessões foram abordadas diferentes habilidades por meio de atividades lúdicas. Todas as sessões foram iniciadas e finalizadas com estimulação auditiva por meio de bombardeamento auditivo. Na sessão 1 foram realizadas atividades que envolvem o reforço articulatório do som alvo. Nas sessões 2 e 3 foram realizadas atividades para a discriminação auditiva e consciência fonológica, sempre com oportunidades para a criança treinar a produção do som alvo. Na sessão 4 iniciou-se a abordagem do som alvo 2 do processo fonológico 1 que estava sendo abordado desde a sessão 1, repetindo nas sessões 4, 5 e 6 os mesmos objetivos das sessões 1, 2 e 3. Na sessão 7 iniciou-se a abordagem do processo fonológico 2, com o primeiro som alvo que apresente as características do mesmo, e seguiu a mesma estrutura detalhada das sessões 1, 2 e 3. Na sessão 10 iniciou-se o segundo som alvo do processo fonológico 2, repetindo-se as abordagens detalhadas acima, também nas sessões 11 e 12. Simplificando, as sessões 1, 4, 7, 10; 2, 5, 8, 11; 3, 6, 9, 12 têm estruturas semelhantes (Apêndice B).

O tempo de duração de cada sessão foi de 50 minutos e sua gestão, conforme detalhado no Quadro 1, permitiu criar e fortalecer o vínculo com a criança, contemplar todas as habilidades a serem abordadas e orientar os pais ao final de cada teleconsulta.

**Quadro 1:** Organização dos objetivos e estratégias terapêuticas das sessões do Pifate

**Sessões**	**Objetivos**	**Duração (m)**	**Estratégias**
1, 4, 7, 10	Estabelecer vínculo	5	Breve conversa aberta com a criança
	Estimulação auditiva	3	Bombardeamento auditivo
	Colocação do som alvo	7	Pistas visuais e fonéticas do TESF
	Reforço articulatório	10	Estratégia 1 pré-definida WordWall^®^
		12	Estratégia 2 a criança escolhe no Pink Cat Games^®^
	Estimulação auditiva	3	Bombardeamento auditivo
	Orientação ao cuidador	10	Observações da sessão e orientações sobre o treino a fazer
2, 5, 8, 11	Estabelecer vínculo	5	Breve conversa aberta com a criança
	Estimulação auditiva	3	Bombardeamento auditivo
	Apresentação dos pares mínimos	10	Jogo WordWall^®^ conduzindo a criança para
		10	classificar os pares mínimos.
	Discriminação auditiva	10	Estratégia 1 pré-definida WordWall^®^
			Estratégia 2 a criança escolhe no Pink Cat Games^®^
	Estimulação auditiva	3	Bombardeamento auditivo
	Orientação ao cuidador	10	Observações da sessão e orientações sobre o treino a fazer
3, 6, 9, 12	Estabelecer vínculo	5	Breve conversa aberta com a criança
	Estimulação auditiva	3	Bombardeamento auditivo
	Fortalecimento da regra fonológica	12	Estratégia 1 pré-definida WordWall^®^
		12	Estratégia 2 a criança escolhe no Pink Cat Games^®^
	Estimulação auditiva	3	Bombardeamento auditivo
	Orientação ao cuidador	10	Observações da sessão e orientações sobre o treino a fazer

Todas as sessões foram realizadas por meio de videochamada utilizando o *Google Meet*, gravadas e salvas de forma a garantir a segurança dos dados. A fonoaudióloga em ambiente privativo, bem iluminado e com luz direta em seu rosto, posicionada de frente para a câmera de um *laptop* com processador 1,6 GHz Intel Core i5 Dual-Core (MacBook), usando fone de ouvido *Blackwire* 3220 (*Plantronics*) com microfone unidirecional, conectado à internet com velocidade de download e de upload acima de 570 Mbps para assim garantir melhor qualidade transmissão de imagem e som. Os pais eram orientados a posicionar a criança sentada, com apoio nos pés, em ambiente silencioso e bem iluminado, preferencialmente usando um tablet ou computador (por possuírem telas maiores) e fones de ouvidos. Os recursos lúdicos utilizados durante as sessões e enviados para o treino diário foram elaborados nas plataformas *WordWall* ^®^ e *Pink Cat Games* ^®^.

### Orientação parental para G1 e G2

Ao final de cada sessão a pesquisadora enviava orientações por e-mail ou *WhatsApp* (um conhecido aplicativo de mensagens instantâneas) de acordo com a preferência do cuidador. As mensagens referentes ao treino a ser realizado em casa após a sessão conduzida pela fonoaudióloga eram semelhantes para ambos os grupos, conforme especificado no Quadro 2.

**Quadro 2:** Orientações parental para os treinos diários em casa (G1 e G2) e para a sessão parental (G2)

**CONTEÚDO DA MENSAGEM DE ORIENTAÇÃO**		**TREINO DIÁRIO G1 E G2**	**SESSÃO PARENTAL G2**
**Manejo do ambiente:**	Controle o ruído do local.	X	X
	Ambiente bem iluminado	X	X
**Ergonomia:**	Criança sentada, com pés apoiados e o dispositivo eletrônico sobre a mesa	X	X
**Equipamentos:**	Prefira dispositivo eletrônico com maior tela possível.	X	X
	Use fones de ouvido durante o bombardeamento auditivo		X
**Treino:**	Treine diariamente	X	
	Saiba mais sobre o som alvo do treino (material enviado aos pais)	X	
	Saiba mais sobre o objetivo do treino e de cada jogo	X	
	Conheça os nomes das figuras utilizadas em cada um dos jogos (material enviado aos pais)	X	
	Leia e/ou veja o tutorial sobre como jogar cada jogo compartilhado	X	
	Envie a gravação em vídeo de trecho do treino à fonoaudióloga	X	
	Envie dúvidas, se tiver, à fonoaudióloga	X	
**Manejo do tempo**	Controle o tempo de duração da sessão (30 a 40 m.)		X
	Conduza a sessão com calma, sem apressar a criança.		X
	Grave vídeos de trechos da sessão		X
**Modelo do padrão articulatório**	Posicione-se em frente da criança para dar o modelo articulatório do som alvo		X
	Fale lentamente e dê destaque ao som alvo		X
	Use as pistas táteis ensinadas		X
**Sessão de intervenção**	Estimulação auditiva: faça o bombardeamento auditivo utilizando áudio gravado e enviado no e-mail		X
	Apresentação dos pares mínimos: nomeie as figuras de cada par (use o gabarito) com a criança		X
	Abra o link e acesse os jogos que serão as estratégias para o que queremos estimular hoje. Leia o tutorial escrito e/ou veja o vídeo sobre como acessar e jogar		X
	Finalize a sessão com o bombardeamento auditivo igual ao do início da sessão		X
	Grave trechos da sessão em vídeo e envie		X
	Envie suas dúvidas sobre a sessão realizada		X
	Aguarde o próximo e-mail com o treino diário a ser realizado até a próxima sessão		X
**Pós-treino:**	Envie os vídeos dos treinos feitos antes da próxima sessão	X	
	Responda o formulário contando o que você achou do interesse, facilidade/dificuldade na execução da tarefa pela criança, também o que você achou do resultado da fala da criança	X	

Aos pais integrantes do G2, somente após este retorno deles foram enviadas orientações e atividades da sessão que eles realizariam em casa sem a participação síncrona da fonoaudióloga. Após a execução da sessão, o cuidador enviava nova amostra de vídeo da sessão para a fonoaudióloga, que então preparava o e-mail do treino, baseado na análise do vídeo.

### Orientação para a intervenção conduzida pelo cuidador do G2

As sessões 1, 4, 6, 7, 10 e 12 foram síncronas com a fonoaudióloga e as sessões 2, 3, 5, 8, 9 e 11 conduzidas pelo cuidador previamente orientado.

Na sessão 1, a fonoaudióloga realizou a intervenção com a criança sob observação do cuidador. Ao final da sessão reservou-se aproximadamente 20 minutos para esclarecimento de dúvidas e treinamento para aplicação do bombardeamento auditivo, treino articulatório, correção da produção articulatória incorreta e reforço positivo da produção correta (Apêndice C).

A mensagem de entrega e orientação quanto ao treino diário em casa foi semelhante à mensagem entregue ao G1. Após as famílias enviarem os vídeos de trechos dos treinos realizados com a criança em casa, juntamente com a entrevista online descrevendo a percepção que tiveram quanto ao treino diário realizado, foi enviada a mensagem referente à sessão 2, contendo agora as estratégias da sessão para condução parental, detalhando como deveria ser a sessão realizada em casa e contemplando os temas apresentados no Quadro 2.

### Análise dos dados

Foi utilizada análise descritiva para caracterização da amostra. A avaliação da diferença entre os grupos, quanto à idade foi realizada utilizando o teste exato de Fisher, e quanto ao sexo, utilizando o teste Mann-Whitney com nível de significância de 0,05.

As respostas da entrevista inicial quanto ao motivo de uso do recurso digital pela criança e o seu tempo de uso foram comparadas entre os grupos aplicando o teste exato de Fischer e Mann Witney, respectivamente, com nível de significância de 5%.

Para conhecer a gravidade do TSF foi realizada a análise da linha de base múltipla (Avaliações A1-A e A1-B) quanto aos índices de PCC, PCC-R, PDI e a ocorrência de processos fonológicos. A comparação entre os indivíduos e entre os grupos foi pelo teste Mann-Whitney, com valor de significância de 0,05.

A eficácia da intervenção foi analisada a partir da medida do tamanho do efeito, calculado considerando as respostas individuais de cada variável: número de diferentes processos fonológicos com mais de 25% de ocorrência na prova de imitação; número de diferentes processos fonológicos com mais de 25% de ocorrência na prova de nomeação; PCC e PCC-R obtidos na prova de imitação; PCC e PCC-R obtidos na prova de nomeação; PDI obtido na prova de imitação; e PDI obtido na prova de nomeação.

Assim, o tamanho do efeito de cada variável foi obtido por meio da diferença da média do resultado observado entre a linha de base múltipla, pré-intervenção, com a média do resultado observado entre as avaliações pós-intervenção, dividido pela variabilidade (desvio padrão) obtido entre os resultados linha de base múltipla pré-intervenção (A1-A e A1-B). Quando os valores pós-intervenção foram menores do que pré-intervenção, o resultado foi apontado com sinal negativo “-”, por exemplo nas variáveis PDI e Produtividade Média dos Processos Fonológicos. O tamanho do efeito pode ser classificado como pequeno, médio e grande^(26)^.

As variáveis da avaliação da percepção dos pais sobre o treino de fala realizado em casa com a criança, que os pais preenchiam após o treino realizado em casa, foram comparadas entre os grupos por meio do teste de Mann-Whitney com nível de significância de 0,05.

## RESULTADOS

O G1 foi composto por cinco crianças do sexo masculino, com idade entre 5:0 e 7:2 anos com gravidade do TSF variando de leve a moderadamente grave e o G2 por três crianças do sexo masculino e duas crianças do sexo feminino, com idade entre 5:2 e 7:7 anos e gravidade do TSF variando de leve a levemente moderada, não havendo diferença estatisticamente significante entre os grupos (sexo p-valor: 0,444; idade p-valor: 0,344; gravidade do TSF p-valor: >0,999).

### Comparação entre G1 e G2 na A1

A comparação entre os dois grupos na A1 não apresentou diferença estatisticamente significante quanto ao número de processos fonológicos com ocorrência nas provas de imitação (p-valor=0,114) e nomeação (p-valor=0,662). O mesmo ocorreu para os valores de PCC (p-valor=0,421), PCC-R (p-valor=0,421) e PDI (p-valor=0,402) na prova de imitação, bem como na prova de nomeação (PCC: p-valor=0,753; PCC-R: p-valor=0,833; PDI: p-valor=0,675).

### Letramento digital da família

Todas as famílias apresentavam conexão com a internet por meio de wi-fi. Todas as crianças tinham acesso controlado à internet, com o objetivo de assistirem vídeos e jogar jogos digitais. Apesar de não apresentar diferença estatisticamente significante entre os grupos quanto ao uso de dispositivos eletrônicos conectados à internet, observou-se maior tempo de uso diário do dispositivo no G1 (41 a 60 minutos, e G2= 21 a 30 minutos. p-valor=0,594).

### Descrição do desempenho de cada sujeito para as variáveis estudadas

Foi calculada a medida do tamanho do efeito^(26)^ considerando as variáveis: número de processos fonológicos com ocorrência maior que 25% nas provas de imitação e nomeação; PCC e PCC-R obtidos nas provas de imitação e nomeação; PDI obtidos na prova de imitação e nomeação.

Na Figura 1 observa-se a ocorrência de cada processo fonológico observado na prova de imitação nas avaliações A1-A, A1-B, A2-A e A2-B para cada criança, e na Tabela 1 encontram-se os resultados médios observados para cada um dos processos antes e após a intervenção, bem como o tamanho de efeito para cada processo para cada criança.

**Figura 1:** Porcentagem de ocorrência de cada processo fonológico observado na prova de imitação nas avaliações A1-A, A1-B, A2-A e A2-B para cada criança

**Tabela 1:** Ocorrência média dos processos fonológicos observados na prova de imitação pré e pós-intervenção e tamanho do efeito para cada processo fonológico em cada criança

	**Ocorrência média de processo fonológico pré-intervenção**	**Ocorrência média de processo fonológico pós-intervenção**	**Tamanho do Efeito**	**Média do Tamanho do Efeito**	**Classificação do Tamanho do Efeito**
*Criança 1*					
FP	88,88%	8,34%	-8,361	-3,167	Médio
SL	80,71%	35,71%	-2,069		
SEC	88,67%	42,26%	-2,364		
SCF	33,33%	15,48%	-2,166		
EF	14,81%	0,00%	-0,873		
*Criança 2*					
FP	66,67%	16,67%	-1,191	-1,191	Pequeno
*Criança 3*					
SL	38,09%	0,00%	-4,620	-4,630	Médio
SEC	58,33%	0,00%	-3,499		
SCF	47,61%	0,00%	-5,771		
*Criança 4*					
SL	49,98%	42,86%	-0,707	-0,234	Pequeno
SEC	62,49%	58,33%	-0,702		
SCF	42,86%	42,86%	0,707		
*Criança 5*					
SL	35,72%	0,00%	-3,535	-3,064	Médio
SEC	70,83%	25,00%	-2,593		
*Criança 6*					
SL	50,00%	2,33%	-2,073	-0,873	Pequeno
SEC	28,57%	21,43%	-0,311		
SCF	16,67%	11,11%	-0,236		
*Criança 7*					
FP	100,00%	25,00%	-5,357	-5,357	Médio
*Criança 8*					
SL	42,86%	0,00%	-5,364	-5,554	Médio
SEC	70,84%	12,50%	-9,904		
SCF	35,71%	21,64%	-1,393		
*Criança 9*					
EP	50,00%	22,82%	-3,459	-6,186	Médio
EF	83,33%	13,25%	-8,913		
*Criança 10*					
FP	91,67%	8,34%	-7,069	-7,069	Grande

Legenda: %: porcentagem; EF: ensurdecimento de fricativa; EP: ensurdecimento de plosiva; FP: frontalização de palatal; SCF: simplificação de consoante final; SEC: simplificação de encontro consonantal; SL: simplificação de líquida

Para comparar o tamanho do efeito entre os grupos, considerando a variabilidade de processos observados em cada criança, foi calculada a média do tamanho do efeito de todos os processos fonológicos produzidos pela criança. As médias obtidas para cada criança foram comparadas entre os grupos por meio do teste Mann-Whitney. Observou-se tendência à significância estatística, sendo que o G2 apresentou maior tamanho de efeito do que o G1 (Tabela 2).

**Tabela 2:** Comparação entre G1 e G2 quanto à média do tamanho do efeito na análise do número de diferentes processos fonológicos com ocorrência >25% observados na prova de imitação

**Grupo**	**Mínimo**	**Máximo**	**Mediana**	**Média**	**Desvio padrão**	**Teste Mann-Whitney**	**p-valor**
Grupo 1	-4,630	-0,234	-3,064	-2,457	1,742	21,000	0,095#
Grupo 2	-7,069	-0,873	-5,554	-5,008	2,406		

# p-valor com tendência à significância estatística

### Número de Diferentes Processos Fonológicos com Ocorrência Maior que 25% na Prova de Nomeação na Linha de Base Múltipla e Reavaliações

Na Figura 2, verifica-se a ocorrência de cada processo fonológico observado na prova de nomeação em cada uma das avaliações A1-A, A1-B, A2-A e A2-B para cada criança, e na Tabela 3 encontram-se os resultados médios observados para cada um dos processos antes e após a intervenção, bem como o tamanho de efeito para cada processo para cada criança.

**Figura 2:** Porcentagem de ocorrência de cada processo fonológico observado na prova de nomeação para cada criança nas avaliações A1-A, A1-B, A2-A e A2-B

**Tabela 3:** Ocorrência média dos processos fonológicos observados na prova de nomeação pré e pós-intervenção e tamanho do efeito para cada processo fonológico em cada criança

	**Ocorrência média de processo fonológico pré-intervenção**	**Ocorrência média de processo fonológico pós-intervenção**	**Tamanho do Efeito**	**Média do Tamanho do efeito**	**Classificação do Tamanho do Efeito**
*Criança 1*					
FP	93,33%	10,00%	-7,217	-4,063	Médio
SL	72,72%	13,55%	-6,513		
SEC	70,83%	31,25%	-1,521		
SCF	60,00%	40,00%	-1,000		
*Criança 2*					
FP	30,00%	0,00%	-0,707	-0,707	Pequeno
*Criança 3*					
SL	44,45%	0,00%	-25,663	-11,967	Grande
SEC	66,67%	0,00%	-9,238		
SCF	40,00%	20,00%	-1,000		
*Criança 4*					
SL	54,54%	50,00%	-0,853	-1,066	Pequeno
SEC	87,50%	75,00%	-2,345		
SCF	60,00%	60,00%	0,000		
*Criança 5*					
SL	45,45%	4,55%	-4,633	-4,423	Médio
SEC	68,75%	31,50%	-4,214		
*Criança 6*					
SL	81,25%	31,25%	-5,657	-0,273	Pequeno
SEC	60,00%	30,00%	-2,308		
SCF	23,41%	16,67%	-0,388		
*Criança 7*					
FP	70,00%	0,00%	-4,950	-4,950	Médio
*Criança 8*					
SL	40,91%	0,00%	-6,364	-5,588	Médio
SEC	93,75%	25,00%	-7,778		
SCF	60,00%	40,00%	-2,621		
*Criança 9*					
EP	39,28%	21,29%	-0,712	-4,242	Médio
EF	61,11%	0,00%	-7,772		
*Criança 10*					
FP	90,00%	0,00%	-6,364	-3,889	Médio
SCF	30,00%	10,00%	-1,414		

Legenda: %: porcentagem; EF: ensurdecimento de fricativa; EP: ensurdecimento de plosiva; FP: frontalização de palatal;

SCF: simplificação de consoante final; SEC: simplificação de encontro consonantal; SL: simplificação de líquida

Para comparar o tamanho do efeito entre os grupos, considerando a variabilidade de processos observados em cada criança, foi calculada a média do tamanho do efeito de todos os processos fonológicos produzidos pela criança e as médias obtidas para cada criança comparadas entre os grupos por meio do teste Mann-Whitney. Não foi observada diferença significante entre os grupos sugerindo uma similaridade na eficácia do tratamento independente do protocolo terapêutico (Tabela 4).

**Tabela 4:** Comparação entre os grupos quanto ao número de diferentes processos fonológicos com ocorrência >25% observados na prova de nomeação

**Grupo**	**Mínimo**	**Máximo**	**Mediana**	**Média**	**Desvio padrão**	**Teste Mann-Whitney**	**p-valor**
Grupo 1	-11,967	-0,707	-4,063	-4,445	4,531	13,000	>0,999
Grupo 2	-5,588	-0,273	-4,242	-3,788	2,071		

### PCC e PCC-R nas provas de imitação e nomeação na linha de base múltipla e reavaliações

Na Figura 3, encontram-se os valores encontra-se o valor de PCC e PCC-R obtidos tanto na prova de imitação quanto na nomeação em cada uma das avaliações A1-A, A1-B, A2-A e A2-B para cada criança.

**Figura 3:** Valores do PCC e PCC-R obtidos nas provas de imitação e nomeação para cada criança nas avaliações A1-A, A1-B, A2-A e A2-B

A classificação do tamanho do efeito em relação aos valores médios de PCC e PCC-R na prova de imitação pré e pós-intervenção variou de pequeno à grande, tanto para os sujeitos do G1 quanto para os sujeitos do G2. Na comparação do tamanho do efeito entre os grupos, não foi observada diferença significante sugerindo uma similaridade na eficácia do tratamento independente do protocolo terapêutico (Tabela 5).

**Tabela 5:** Comparação do tamanho do efeito entre G1 e G2 para os valores de PCC e PCC-R na prova de imitação e de nomeação

	**Mínimo**	**Máximo**	**Mediana**	**Média**	**Desvio padrão**	**Teste Mann-Whitney**	**p-valor**
*PCC Imitação*							
Grupo 1	0,667	15,588	1,717	4,331	6,362	6,000	0,222
Grupo 2	0,988	19,108	4,235	6,973	7,071		
*PCC-R Imitação*							
Grupo 1	0,881	17,484	3,085	5,351	6,927	6,000	0,222
Grupo 2	3,216	9,710	5,657	6,224	2,436		
*PCC Nomeação*						13,000	>0,999
Grupo 1	-0,350	18,968	5,734	6,153	7,738		
Grupo 2	0,764	6,541	4,300	3,426	2,462		
*PCC-R Nomeação*							
Grupo 1	0,707	20,626	5,300	7,963	8,436	12,000	>0,999
Grupo 2	1,393	17,163	4,654	6,433	6,444		

### PDI nas provas de imitação e nomeação na linha de base múltipla e reavaliações

Na Figura 4 encontra-se o valor de PDI obtido tanto na prova de imitação quanto na nomeação em cada uma das avaliações A1-A, A1-B, A2-A e A2-B para cada criança.

**Figura 4:** Valor de PDI obtido nas provas de imitação e nomeação para cada criança nas avaliações A1-A, A1-B, A2-A e A2-B

Assim como na classificação do tamanho do efeito em relação aos valores médios de PCC e PCC-R, a comparação do tamanho do efeito de PDI na prova de imitação pré e pós-intervenção variou de pequeno à grande tanto para os sujeitos do G1 quanto para os sujeitos do G2, não apresentando diferença estatisticamente significante.

### Percepção dos pais sobre o treino de fala realizado em casa com a criança

O tempo de interesse da criança por cada uma das duas atividades de treino foi em média de 11 a 15 minutos em ambos os grupos, sem diferença significativa entre os grupos pelo teste de Mann-Whitney (p-valor> 0,999).

De acordo com a avaliação dos pais de ambos os grupos, as crianças tiveram grande interesse pelas atividades propostas para o treino em casa, sinalizando uma média de resposta de 4 pontos em uma escala Likert de 5 pontos, sem diferença significativa entre os grupos pelo teste de Mann-Whitney (p-valor=0,600) (Figura 5).

**Figura 5:** Média das respostas referentes à percepção das famílias quanto aos treinos diários

As crianças foram orientadas pelos pais quanto à forma de treinar em casa o som alvo estimulado. De acordo com os pais, elas tiveram facilidade na compreensão destas orientações, alcançando uma pontuação média obtida neste questionamento de 4 pontos em ambos os grupos, precisando de pouco apoio para a realização do treino, com pontuação média de 2 pontos para ambos os grupos. A comparação dos grupos para ambas as perguntas não apresentou diferença significativa entre eles pelo teste de Mann-Whitney (p-valor=0,600 e p-valor=0,911) (Figura 5).

Ao ser questionada sobre como foi realizar o treino de fala com a criança, a resposta média obtida foi de 4 pontos em ambos os grupos, sem diferença significativa entre os grupos pelo teste de Mann-Whitney (p-valor>0,999) (Figura 5).

## DISCUSSÃO

A aplicação da telefonoaudiologia na intervenção dos TSF em população pediátrica é uma solução que possibilita acesso ao tratamento mesmo em situações desafiadoras, como grandes distâncias entre a família e o profissional, dificuldade de locomoção ou, em situações críticas^(1,9,14)^, como ocorreu durante o período da pandemia de covid-19, possibilitando o acompanhamento profissional, mesmo durante os meses de isolamento social^(31)^.

A utilização de jogos digitais sérios com enfoque no aprendizado da fala e as plataformas que oferecem recursos de edição e personalização de tais jogos^(10,32)^ ampliou ainda mais os recursos para a prática clínica online.

A fim de comparar a eficácia das estratégias lúdicas com material físico e as estratégias lúdicas com jogos sérios digitais utilizados em sessão presencial na intervenção de crianças com TSF, pesquisadores^(33,34)^ verificaram que ambos os grupos apresentaram melhora da inteligibilidade de fala após a intervenção. Assim, constataram que o uso de jogos digitais como recursos terapêuticos é tão eficaz quanto a abordagem tradicional com recursos físicos em mesa no tratamento de crianças com TSF idiopático.

Os resultados do presente estudo mostraram que o Pifate foi eficaz para todos os sujeitos, com tamanho do efeito variando de pequeno a grande, promovendo a redução da ocorrência dos processos fonológicos (indicada pela comparação de produtividade dos processos fonológicos), e consequente melhora da inteligibilidade de fala (indicada pela comparação de PCC, PCC-R e PDI) para ambos os grupos de intervenção, sem diferença estatisticamente significante entre eles. As variações do tamanho do efeito entre os sujeitos podem estar relacionadas com as diferentes características intrínsecas de cada sujeito. Pesquisadores^(35)^ estudaram o efeito da aplicação das oposições múltiplas no tratamento dos TSF em crianças e analisaram a participação dos pais na entrega da intervenção. Seus achados também foram positivos, com variação do tamanho do efeito entre os sujeitos, indicando, ainda, que os pais devidamente treinados são capazes de conduzir adequadamente a sessão de intervenção fonoaudiológica de seu filho.

Manter os pais engajados durante todo o processo também foi considerado um objetivo terapêutico, independente se integravam o G1 ou o G2. Além de solicitar que os pais acompanhassem todas as sessões conduzidas pela fonoaudióloga, garantindo assim aprendizado e maior aptidão para aplicação do treino com a criança, também foram preparados materiais de orientação para o treino de todas as sessões, com textos simples e exemplos do que lhe era solicitado aplicar com as crianças. De acordo com estudo recente^(36)^, a comunicação orientada e informativa do fonoaudiólogo com os pais garante maior engajamento dos mesmos na realização do treino diário, sendo muitas vezes necessário mudar as estratégias de orientação de acordo com as características de cada cuidador.

Pesquisadores^(37)^ consideram que há ainda uma cultura do atendimento em saúde ser presencial, sendo importante considerar o treinamento tanto dos profissionais quanto da criança e pais, para melhor aproveitamento dos serviços oferecidos por meio da telefonoaudiologia. O acompanhamento das estratégias aplicadas pelos pais foi feito por meio do compartilhamento de vídeos de parte dos treinos e sessões realizadas em casa com a criança, bem como o preenchimento de um formulário online com questões estimulando uma análise crítica do treino realizado em casa. Assim a fonoaudióloga pôde orientar os pais, ajustar detalhes e palavras de elogio como incentivo para que continuassem ou melhorassem nos treinos das sessões seguintes.

Conhecer o perfil de letramento digital foi importante para garantir que grandes diferenças deste aspecto entre os sujeitos não interferissem nos resultados alcançados. A díade cuidador-criança de G1 e G2 apresentaram perfil de letramento sem diferença estatisticamente significante. Porém notou-se que as crianças do G1 faziam um de dispositivo eletrônico mais tempo durante o dia. O perfil socioeconômico e escolaridade dos pais não foram investigados neste estudo, não sendo possível considerá-los nas hipóteses ao longo da discussão.

O engajamento dos pais foi fundamental para os resultados alcançados pelas crianças de ambos os grupos. O tamanho do efeito^(26)^ da intervenção nos sujeitos da pesquisa, variou de pequeno a grande, sendo a diferença entre os grupos com tendência à significância estatística apontando maior tamanho do efeito no G2. Mesmo sendo apenas uma tendência, que poderia ser confirmada ou não caso o estudo prosseguisse com a captação de mais sujeitos em cada grupo, é interessante apontar que o grupo com menos sessões síncronas com a fonoaudióloga teve tendência a maiores valores de tamanho do efeito em relação ao grupo que realizou toda as sessões síncronas com a profissional. Uma hipótese para explicar tal tendência à diferença estatística é que quando os pais estão mais envolvidos no processo terapêutico eles podem apresentar melhor aproveitamento nos treinos diários com a criança, bem como inserindo estratégias de correção e produção de fala nas atividades cotidianas da criança, gerando maior assimilação e automatização do som alvo pela criança^(20)^.

Ao analisarem^(38)^ a percepção dos pais quanto à experiência com a telefonoaudiologia no tratamento de seus filhos, observou-se que os pais valorizaram o papel ativo que desempenharam no processo de terapia de seus filhos, apesar dos desafios práticos e emocionais associados à telefonoaudiologia, apontando também a importância de mensurar o nível de complexidade dos treinos de fala solicitados aos pais, e entregar atividades que os pais consideram como fáceis de serem realizadas com as crianças pois estas tem maior aceite pelos mesmos.

Por mais que a dose e a frequência dos estímulos oferecidos pela fonoaudióloga, tenham sido semelhantes para ambos os grupos, pode-se considerar a hipótese de que as famílias do G2 ofereceram espontaneamente o treino diário e as estratégias das sessões em uma frequência maior, aumentando assim a intensidade cumulativa da intervenção^(39)^ e, consequentemente, uma tendência ao maior tamanho do efeito do Pifate neste grupo.

Entretanto é importante destacar a limitação do estudo devido ao tamanho amostral reduzido, o que dificultou a análise estatística na comparação entre os grupos. Assim, para que se confirme a tendência à diferença estatisticamente significante de melhores resultados no grupo 2 em relação ao grupo 1, se faz necessária a continuidade do estudo com o aumento do número de sujeitos de ambos os grupos.

## CONCLUSÃO

O estudo realizado demonstrou a eficácia do Pifate, evidenciando que é possível a aplicação da abordagem de intervenção dos Ciclos adaptada por meio de telefonoaudiologia.

O desempenho dos dois grupos foi semelhante, sugerindo que a aplicação de uma sessão síncrona com a fonoaudióloga por telefonoaudiologia e uma pelos pais previamente orientados pela fonoaudióloga resultou em melhora na fala da criança. Destaca-se que a orientação e o treinamento dos pais pela fonoaudióloga por telefonoaudiologia foi fundamental para proporcionar bom engajamento na aplicação das sessões e nas tarefas diárias em casa.
